# Induction of labour with a Foley catheter or oral misoprostol at term: the PROBAAT-II study, a multicentre randomised controlled trial

**DOI:** 10.1186/1471-2393-13-67

**Published:** 2013-03-19

**Authors:** Mieke LG ten Eikelder, Femke Neervoort, Katrien Oude Rengerink, Marta Jozwiak, Jan-Willem de Leeuw, Irene de Graaf, Maria G van Pampus, Maureen Franssen, Martijn Oudijk, Paulien van der Salm, Mallory Woiski, Paula JM Pernet, A Hanneke Feitsma, Huib van Vliet, Martina Porath, Frans Roumen, Erik van Beek, Hans Versendaal, Marion Heres, Ben Willem J Mol, Kitty W M Bloemenkamp

**Affiliations:** 1 Department of Obstetrics K-6-P-35, Leiden University Medical Centre, P.O. Box 9600, Leiden 2300 RC, the Netherlands; 2 Department of Obstetrics and Gynaecology, Onze Lieve Vrouwe Gasthuis, Amsterdam, the Netherlands; 3 Department of Obstetrics and Gynaecology, Academic Medical Centre, Amsterdam, the Netherlands; 4 Department of Obstetrics and Gynaecology, Ikazia Hospital, Rotterdam, the Netherlands; 5 Department of Obstetrics and Gynaecology, University Medical Centre Groningen, Groningen, the Netherlands; 6 Department of Obstetrics and Gynaecology, Radboud University, Nijmegen, the Netherlands; 7 Department of Obstetrics and Gynaecology, University Medical Centre Utrecht, Utrecht, the Netherlands; 8 Department of Obstetrics and Gynaecology, Kennemer Gasthuis, Haarlem, the Netherlands; 9 Department of Obstetrics and Gynaecology, HAGA Hospital, Den Haag, the Netherlands; 10 Department of Obstetrics and Gynaecology, Catharina Hospital, Eindhoven, the Netherlands; 11 Department of Obstetrics and Gynaecology, Maxima Medical Centre, Veldhoven, the Netherlands; 12 Department of Obstetrics and Gynaecology, Atrium Medical Centre, Heerlen, the Netherlands; 13 Department of Obstetrics and Gynaecology, Sint Antonius Hospital, Nieuwegein, the Netherlands; 14 Department of Obstetrics and Gynaecology, Maasstad Hospital, Rotterdam, the Netherlands; 15 Department of Obstetrics and Gynaecology, Sint Lucas Andreas Hospital, Amsterdam, the Netherlands

**Keywords:** Induction of labour, Oral misoprostol, Foley catheter, Asphyxia, Post partum haemorrhage, Hyperstimulation, Bishop score, Unfavourable cervix

## Abstract

**Background:**

Induction of labour is a common obstetric procedure. At present, different methods are used for induction of labour in women with an unfavourable cervix. Recently, we showed that in term women with an unfavorable cervix the use of a Foley catheter in comparison with vaginal Prostaglandin E2 gel, results in a comparable vaginal delivery rate. A meta-analysis on the subject indicated lower rates of hyperstimulation, and probably as a sequel fewer cases of postpartum haemorrhage. Misoprostol (PgE1) is another type of prostaglandin frequently used for labour induction, recommended by the international federation of gynaecology and obstetrics (FIGO). Misoprostol can be administered by vaginal, rectal and oral route. There is evidence that oral administration results in less asphyxia and hyperstimulation than vaginal administration. At present, valid comparisons between oral misoprostol and Foley catheter are lacking. Therefore, we propose a randomised controlled trial comparing Foley catheter to oral misoprostol in order to assess safety and cost-effectiveness.

**Methods/Design:**

We plan a multicentre, randomised, controlled, open-label clinical trial among term pregnant women with a vital singleton in cephalic presentation, unfavorable cervix, intact membranes and an indication for induction of labour. After informed consent, women will be randomly allocated by a webbased randomisation system to transcervical Foley catheter or oral misoprostol (50 mcg every 4 hours). The primary outcome will be a composite of complications of uterine hyperstimulation, i.e. post partum haemorrhage and asphyxia. Secondary outcomes are mode of delivery, maternal and neonatal morbidity, costs and women’s preference. Serious adverse events such as severe maternal or neonatal morbitity or mortality will be monitored and reported to an independent data safety monitory board. With a sample size of 1860 women we will be able to demonstrate a 5% non-inferiority of the Foley catheter as compared to misoprostol for the composite outcome.

**Discussion:**

Worldwide, various methods are being used for labour induction. Results of the proposed trial will contribute to the answer which method of induction of labour is most safe, cost-effective, and patient friendly and will help to construct evidence based guidelines.

**Trial registration:**

The Netherlands Trial Register NTR3466

## Background

Induction of labour is a commonly practiced obstetric intervention designed to artificially initiate the process of effacement of the cervix, dilatation (cervical ripening), uterine contractions (often after augmentation with oxytocin) and eventually delivery of the baby. Common indications for induction of labour are post-term pregnancy, hypertensive disorders, diabetes in pregnancy, cholestasis, decreased physical activity of the fetus and prelabour rupture of membranes. Induction of labour with an unfavorable cervix is associated with prolonged labour compared to spontaneous onset of labour or induction of labour with a favorable cervix. Also an increase in instrumental deliveries and a higher rate of caesarean sections are seen [1-3]. To increase the success of labour induction it is essential to achieve cervical ripening in women with an unfavorable cervix. Misoprostol has been widely studied as an agent for induction of labour. There are currently two Cochrane reviews focusing on the use of both oral and vaginal misoprostol [4,5]. These systematic reviews conclude that misoprostol is an effective and safe induction agent. Oral misoprostol is preferred to vaginal administration for as it shows lower rates of uterine hyperstimulation and adverse neonatal outcome [4,5]. Although not licensed for induction of labour, misoprostol is recommended by several guidelines. The British Royal College of Obstetricians and Gynaecologists (RCOG), as well as the World Health Organization (WHO), recommend the use of misoprostol tablets, based on cost-effectiveness. In the Dutch Society of Gynaecology and Obstetrics (NVOG) guideline for induction of labor, misoprostol is included and reported to be effective and safe [6-8]. Another method for induction of labour is the transcervical Foley catheter. We recently showed (PROBAAT trial) that induction of labour with a Foley catheter is as effective as induction with intravaginal prostaglandin E2 gel, with fewer maternal and neonatal side-effects [9]. The caesarean section rate was comparable and in the meta-analysis of three trials on the subject, the Foley catheter revealed a lower rate of hyperstimulation, resulting in fewer cases of asphyxia and less post partum haemorrhage. Consequently, the transcervical Foley catheter was recommended for induction of labour in women with an unfavorable cervix at term [9]. The Foley catheter is reported to have similar success rates as induction of labour with misoprostol (vaginal and oral), and is associated with less uterine hyperstimulation with and without fetal heart rate (FHR) changes and a comparable caesarean section rate [10-14]. As prostaglandins in general have been shown to cause an increased incidence of hyperstimulation, we expect a decreased blood flow of the placenta and consequently an increased the risk of asphyxia. In addition, hyperstimulation might lead to uterine muscle fatigue and thereby increase the risk for post partum haemorrhage. However, randomised controlled trials currently available are underpowered to investigate these estimators. In view of the scarce evidence on the subject, we propose a adequately-powered randomised controlled trial comparing induction of labour with a Foley catheter to induction of labour with oral misoprostol in women with an unfavorable cervix at term. In this trial the safety, cost-effectiveness and patient preference will be evaluated.

## Methods/Design

### Aims

The aim of this study is to assess the safety and cost-effectiveness of induction of labour with a transcervical Foley catheter as compared to induction with oral misoprostol in term pregnant women with an unfavourable cervix.

### Participants/eligibility criteria

Term pregnant women will be informed about the trial at the moment the decision is made to induce labour. Eligible are women ≥ 18 years with a gestational age ≥37 weeks with a vital singleton in cephalic presentation, intact membranes and an unfavourable cervix (Bishop score <6). Exclusion criteria are hypersensitivity for any of the products used, a history of caesarean section, placenta praevia, or lethal congenital anomalies.

### Procedures, recruitment, randomisation, collection of baseline data

This trial will be a multicentre, open-label, randomised controlled trial. The study will be performed within the Dutch Consortium for Studies in Women’s Health and Reproductivity (http://www.studies-obsgyn.nl). Participating hospitals can be district, teaching or academic hospitals. Before entry into the study, women will be informed about the aims, methods, reasonably anticipated benefits and potential hazards of the study. This will generally be at least 1 day before actual admission for induction. They will be informed that their participation is voluntary and that they may withdraw consent for participation at any time during the study. Choosing not to participate will not affect care. In each centre an independent physician will be available for more detailed information for both patients and colleagues if desired. After counselling, written informed consent will be obtained. The consent form must be signed before performing of any study-related activity. Randomisation will be performed web-based using ALEA. After entering patient initials, confirming inclusion criteria and absence of exclusion criteria, randomisation will be performed realtime based on an algorithm. We will use fixed block sizes of 2 and 4 and stratify for centre and parity. As there is no prespecified randomisation list, it will be hardly possible for anyone to know the next allocation. Randomisation will take place after evaluation of the fetal condition by CTG, just before start of induction. Eligible women will be allocated in a 1:1 ratio to induction with a Foley catheter or oral misoprostol. There will be no blinding of patients and caregivers, as this is not feasible with these two treatment methods. Baseline demographic, obstetric and medical histories, and details of delivery and health care received till the time of discharge will be recorded for all women. Data will be entered into an electronic case-record form (Oracle Clinicle version 4.5.3) accessible through our study website with a personal login http://www.studies-obsgyn.nl/probaat2. All changes made in this electronic case-record form will be captured in an audit trial. In Oracle Clinicle checks will be programmed to identify errors. All electronic case-record forms will be checked for consistency and to correct errors.

### Interventions

At the start of the study, each centre will be instructed in a brief presentation and practical training. When needed, a local research nurse is available during the inclusion period to advise the staff on use of the Foley catheter and/or oral misoprostol.

#### Induction with a transcervical Foley catheter

A 16 or 18 F Foley catheter will be introduced into the cervix and the balloon is filled with 30 cc of 0.9% NaCl or water. The Foley catheter can be placed digitally or using a speculum according to preference of the treating physician. No recommendations regarding disinfection will be given as there is no evidence for the best method. The external end of the Foley catheter will be tapped to the thigh without giving traction. Foley catheter location after placement can be evaluated digitally and/or by ultrasound. Women will be instructed to observe one hour of bed rest after every newly placed Foley catheter, while fetal condition and uterine activity will be monitored. Women will be examined every 12 hours if the transcervical Foley catheter does not detach spontaneously. When the Bishop score remains <6; the location of the Foley catheter will be evaluated and a new Foley catheter will be placed after 24 of 48 our depending on the preference of the participating centre.

#### Induction with oral misoprostol

Patients in the misoprostol group will be treated according to the protocol used in the study of Gemund et al. [15]. Misoprostol will be administered every four hours with a maximum of three times a day for 4 days. For this study the protocol was adjusted to use of oral misoprostol. Women in the misoprostol group will receive 50 mcg misoprostol capsules. Since misoprostol 50 mcg tablets are not on the market and misoprostol as a raw material is not available, the 50 mcg tablets are made from the originally Cytotec 200 mcg, Searle, Maarsen, The Netherlands by the Leiden University Medical Centre pharmacy. 200 mcg Cytotec tablets are pulverized with a cube mixer. Capsules are prepared from portions of the powder mixture. Amount of powder mixture is optionally supplemented with microcrystalline cellulose to achieve volume for 100 capsules. Each capsule contains between 47.5 and 52.5 mcg misoprostol. High-performance liquid chromatography shows the standard retention time and sample are the same (within 2.5%). Women will be instructed to observe one hour of bed rest after every dose of misoprostol, while fetal condition and uterine activity will be monitored. When there will be 3 or more contractions in 10 minutes while the Bishop score is still <6 or when the fetal heart rate is not reassuring the next misoprostol dose will be withheld. Amniotomy and oxytocin infusion can be started at any moment of the day when the Bishop score is ≥ 6, and at least 4 hours after the last dose of misoprostol. Women will be treated for a maximum of 4 days with Foley catheter or oral misoprostol. If after 4 days the cervix remains unfavorable, the induction is considered to be failed and further management will be decided individually by the treating physician. The number of women needing more than 48 hours of induction is expected to be very low.

### Outcome measures

The primary endpoint will be a composite outcome of neonatal asphyxia (defined as a neonatal pH ≤ 7.05 and/or 5 minute Apgar score <7) and/or post partum haemorrhage (defined as an estimated blood loss of ≥1000 cc ascertained over 24 h post partum). We have chosen this combined outcome as both conditions are thought to be a result of hyperstimulation.

Secondary endpoints:

• Mode of delivery (Caesarean section, vaginal operative delivery, spontaneous delivery) and the reason for operative delivery, i.e. suspected fetal distress and/or failure to progress.

• Induction to delivery time,

• Use of analgetics

• Oxytocin use

• Number of misoprostol gifts/Foley catheters used.

• Number of vaginal examinations

• Maternal morbidity

 ○ Post partum blood transfusion and number of packed cells

 ○ Tachysystole (defined as more than five contractions in ten minutes over a minimal period of two times ten minutes)

 ○ Hyperstimulation (defined as tachysystole with FHR changes (defined as a non reassuring CTG by treating physician))

 ○ Uterine hypertonus (defined as a contraction lasting longer than two minutes with FHR changes)

 ○ Uterine rupture (occurrence of clinical symptoms (abdominal pain, abnormal fetal heart rate pattern, acute loss of contractions, vaginal blood loss) leading to an emergency caesarean delivery, at which the presumed diagnosis of uterine rupture was confirmed; or peripartum hysterectomy or laparotomy for uterine rupture after vaginal birth)

 ○ Uterine scar dehiscence (separation of a pre-existing scar that does not disrupt the uterine serosa as seen during caesarean section, without clinical consequences)

 ○ Maternal infection during labour (defined as fever, i.e. temperature ≥ 37.8°C, or fetal tachycardia AND start of antibiotics)

 ○ Maternal infection within one week post partum (defined as fever, i.e. temperature ≥ 37.8°C, AND start of antibiotics)

 ○ Start of intravenous antibiotics

 ○ Endo(myo)metritis or urinary tract infection within one week post partum (proven positive vaginal/urine culture)

 ○ Other medication used during labour such as tocolytics.

• Neonatal parameters consisting of:

 ○ Fetal tachycardia (sustained fetal heart rate above 160 beats per minute)

 ○ Gender

 ○ Weight at birth

 ○ Meconium-stained liquor

 ○ Apgar scores <7 at 1 minute

 ○ Admission to the neonatal ward/NICU and its reason (suspected infection, infection proven by positive culture, other reason admission to medium or intensive care).

An economic analysis will be conducted alongside the PROBAAT-II trial. In order to do so, resource use in different phases of delivery will be collected in the electronic case-record form (eCRF). To our knowledge, no validated questionnaires on women’s preference of induction of labour method are available. Therefore women’s preference for the method of induction will be evaluated by a post partum questionnaire developed for this study, based on a validated questionnaire by Wijma et al. This questionnaire was developed to assess women’s experience of labour [16].

### Follow up of women and infants

Details of admission of women and newborns will be recorded as will maternal and neonatal complications. Long term follow-up is not part of the current study.

### Analysis

This trial is designed as a non-inferiority trial. Primary analysis will be by intention-to-treat. In a comparative trial, where the aim is to decide if two treatments will be different, an intention-to-treat analysis is generally conservative: the inclusion of protocol violators and withdrawals will usually make the results from the two treatment groups more similar. However, for a non inferiority trial this effect is no longer conservative: any blurring of the difference between the treatment groups will increase the chance of declaring non inferiority [17]. We will therefore also perform a per protocol analysis. Relative risks and 95% confidence intervals will be calculated for the relevant outcome measures. We will additionally adjust for the stratified randomization when calculating the relative risk. Differences between categorical variables will be tested with the Chi-square test, or if the expected cell count is below 5 with the Fisher’s exact test. Non-normally distributed continuous variables will be tested with the Mann–Whitney *U* test. Time to delivery will be assessed using Kaplan-Meier analysis. For the economic analysis we plan a cost-minimisation procedure, as we designed a non-inferior trial and expect no differences in outcomes. Resource uses are multiplied by their specific costs, which are estimated according to recent guidelines on costing health care services. When a relevant differences in outcomes will be observed, we will perform a cost-effectiveness analysis, in which we calculate the additional cost per prevented case of fluxus or asphyxia and the cost per prevented Caesarean section. Mean resource use and costs are compared between both arms, if any difference of interest in primary and/or secondary outcomes occurs a cost-effectiveness analysis will be performed. Using an interaction term we will assess whether the difference in effect between misoprostol and Foley catheter is consisted in nulliparous and multiparous women.

### Sample size

To calculate an adequate sample size we searched the literature for comparisons between oral misoprostol and Foley catheter. There is only one study which directly compared induction of labour with oral misoprostol and Foley catheter. This study does not report on our primary outcome measures which makes it insufficient to use for our power calculation [10]. A few studies reported on a comparison of vaginal misoprostol and prostaglandin E2, but different dosages were used and from the reported outcome parameters it was not possible to calculate the composite outcome we use [6]. Therefore we used data from the PROBAAT trial, comparing prostaglandin E2 (prostin) with Foley catheter [9]. In the Probaat trial the incidence of the composite outcome of pH ≤ 7.05 and/or an Apgar < 7 at 5 minutes (at least one known) and/or haemorrhage ≥1000 cc post partum was 12.7% when a Foley catheter was used and 16.4% when Prostaglandin E2 gel was used. We expect the incidence of the composite outcome to be lower when using oral misoprostol than when using prostaglandin E2 but slightly higher than when a Foley catheter is used [5]. We expect that about 25% of umbilical cord pH’s will be missing, while the 5 minute Apgar will be complete in nearly all cases. So, if the umbilical artery pH data will be missing, neonatal outcome will be classified as abnormal if the 5 minute Apgar will be <7 and as normal if the Apgar was ≥ 7. With a power of 80% (1-β) and a one-sided 0.05 risk of type I error (α), we need 1860 participants (930 per group) to demonstrate non-inferiority, i.e. that the absolute difference in the composite outcome will be less than 5% in the misoprostol group compared to the Foley catheter group, when assuming that the composite outcome was 12.7% in the Foley catheter group and 13.7% in the misoprostol group and an 5% exclusion rate from the per protocol analysis. This sample size will also give us more than 80% power to demonstrate non-inferiority, i.e. less than 5% increase in the proportion of caesarean sections when using misoprostol compared to Foley catheter, assuming 23% caesarean sections in both groups [9]. We used NQuery Advisor 7.0, with the two group test of equivalence in proportion (NQueryAdvisor; Machin 1987) to calculate the sample size.

### Monitoring

An independent data and safety monitoring board (DSMB) will be established prior to the start of the trial. All the serious adverse events (SAE’s) (intra uterine fetal death (IUFD), uterine rupture, severe maternal and neonatal morbidity such as IC/NICU admission and related events such as placental abruption directly after insertion of Foley catheter) will be reported within 48 hours to the CCMO (central commission human related research). All SAEs will be reported to the DSMB at 300 and 600 inclusions when the interim analysis will take place, or when in total 5 SAEs are reported, whichever comes first. Prior to the start of the trial the DSMB defined criteria to terminate the trial prematurely. The DSMB will perform 2 interim analyses: after recruitment of 300 and 600 patients. These interim analyses will be conducted independently of the research group, and will take into account safety (infection, asphyxia, haemorrhage) as well as efficacy. The study will be terminated prematurely for efficacy according to the Haybittle rule when there is a difference with P <0.001.

### Ethical considerations

This study has been approved by the National Central Committee on Research involving Human Subjects (CCMO - NL 35278.018.11.), by the ethics committee of the Academical Medical Centre (Ref. No. 2011_010#B2012219). The boards of directors of all participating hospitals approved local execution of the study.

## Discussion

In Western countries, labour is induced in 20-30% of all pregnant women for various reasons. Until now different methods for labour induction are used. In literature contradictory results are reported regarding efficacy and safety of the induction methods. The outcome of our recent study which compared prostaglandin gel to a Foley catheter was that the Foley catheter showed fewer maternal and neonatal side-effects and was as effective as prostaglandin gel [9]. Another widely used method of labour induction is the administration of misoprostol. Misoprostol is recommended by several (inter)national guidelines, based on cost-effectiveness [6,8]. However, randomised controlled trials are underpowered to investigate the estimators of interest. Therefore we will compare in an adequately powered randomised open-label controlled trial induction of labour at term with a Foley catheter to oral misoprostol. Taking into account the possible risks, costs and women’s preferences; this study could assess the safest, most cost-effective and patient friendliest way of inducing labour with an unfavorable cervix. Eventually this study could contribute to achieve consensus in the guidelines, thereby improving the care for over 20% of all the pregnant women in western countries from who labour will be induced.

## Abbreviations

CCMO: Central Committee on Research Involving Human Subjects; in Dutch: Centrale Commissie Mensgebonden Onderzoek; DSMB: Data safety monitoring board; FHR: Fetal heart rate; (S)EA: (Serious) Adverse event.

## Competing interests

The authors declare that they have no competing interests.

## Authors’ contributions

ME, KB, KOD and BWM were involved in the conception and design of the study. ME, KOD and MJ drafted the manuscript. All authors mentioned in the manuscript are members of the PROBAAT-II-trial study group. They are local investigators at the participating centres. All authors read, edited and approved the final manuscript.

## Pre-publication history

The pre-publication history for this paper can be accessed here:

http://www.biomedcentral.com/1471-2393/13/67/prepub
