# Correction: Induction of labour with a Foley catheter or oral misoprostol at term: the PROBAAT-II study, a multicentre randomised controlled trial

**DOI:** 10.1186/1471-2393-13-183

**Published:** 2013-10-31

**Authors:** Mieke LG ten Eikelder, Femke Neervoort, Katrien Oude Rengerink, Gert J van Baaren, Marta Jozwiak, Jan-Willem de Leeuw, Irene de Graaf, Maria G van Pampus, Maureen Franssen, Martijn Oudijk, Paulien van der Salm, Mallory Woiski, Paula JM Pernet, A Hanneke Feitsma, Huib van Vliet, Martina Porath, Frans Roumen, Erik van Beek, Hans Versendaal, Marion Heres, Ben Willem J Mol, Kitty WM Bloemenkamp

**Affiliations:** 1 Department of Obstetrics K-6-P-35, |Leiden University Medical Centre, P.O. Box 9600, Leiden 2300 RC, the Netherlands; 2 Department of Obstetrics and Gynaecology, Onze Lieve Vrouwe Gasthuis, Amsterdam, the Netherlands; 3 Department of Obstetrics and Gynaecology, Academic Medical Centre, Amsterdam, the Netherlands; 4 Department of Obstetrics and Gynaecology, Ikazia Hospital, Rotterdam, the Netherlands; 5 Department of Obstetrics and Gynaecology, University Medical Centre Groningen, Groningen, the Netherlands; 6 Department of Obstetrics and Gynaecology, Radboud University, Nijmegen, the Netherlands; 7 Department of Obstetrics and Gynaecology, University Medical Centre Utrecht, Utrecht, the Netherlands; 8 Department of Obstetrics and Gynaecology, Kennemer Gasthuis, Haarlem, the Netherlands; 9 Department of Obstetrics and Gynaecology, HAGA Hospital, Den Haag, the Netherlands; 10 Department of Obstetrics and Gynaecology, Catharina Hospital, Eindhoven, the Netherlands; 11 Department of Obstetrics and Gynaecology, Maxima Medical Centre, Veldhoven, the Netherlands; 12 Department of Obstetrics and Gynaecology, Atrium Medical Centre, Heerlen, the Netherlands; 13 Department of Obstetrics and Gynaecology, Sint Antonius Hospital, Nieuwegein, the Netherlands; 14 Department of Obstetrics and Gynaecology, Maasstad Hospital, Rotterdam, the Netherlands; 15 Department of Obstetrics and Gynaecology, Sint Lucas Andreas Hospital, Amsterdam, the Netherlands

## 

Additional author: During the editing process of the article “Induction of labour with a Foley catheter or oral misoprostol at term: the PROBAAT-II study, a multicentre randomised controlled trial“ [1] an author has been accidentally removed from the author list. Gert J van Baaren (Department of Obstetrics and Gynecology, Academic Medical Centre, Amsterdam, the Netherlands, g.j.vanbaaren@amc.uva.nl) critically revised the first draft, and approved the final version of the manuscript. He was especially involved in the sections relating to the cost-effectiveness analysis.

## Pre-publication history

The pre-publication history for this paper can be accessed here:

http://www.biomedcentral.com/1471-2393/13/183/prepub
